# Target Enrichment Approaches for Next-Generation Sequencing Applications in Oncology

**DOI:** 10.3390/diagnostics12071539

**Published:** 2022-06-24

**Authors:** Rajesh R. Singh

**Affiliations:** Department of Molecular Oncology, Quest Diagnostics Nichols Institute, Chantilly, VA 20151, USA; rajesh.r.singh@questdiagnostics.com; Tel.: +1-703-802-7151; Fax: +1-703-802-7135

**Keywords:** next-generation sequencing, NGS, target enrichment, polymerase chain reaction, amplicon, hybridization capture

## Abstract

Screening for genomic sequence variants in genes of predictive and prognostic significance is an integral part of precision medicine. Next-generation sequencing (NGS) technologies are progressively becoming platforms of choice to facilitate this, owing to their massively parallel sequencing capability, which can be used to simultaneously screen multiple markers in multiple samples for a variety of variants (single nucleotide and multi nucleotide variants, insertions and deletions, gene copy number variations, and fusions). A crucial step in the workflow of targeted NGS is the enrichment of the genomic regions of interest to be sequenced, against the whole genomic background. This ensures that the NGS effort is focused to predominantly screen target regions of interest with minimal off-target sequencing, making it more accurate and economical. Polymerase chain reaction-based (PCR, or amplicon-based) and hybridization capture-based methodologies are the two prominent approaches employed for target enrichment. This review summarizes the basic principles of target enrichment utilized by these methods, their multiple variations that have evolved over time, automation approaches, overall comparison of their advantages and drawbacks, and commercially available choices for these methodologies.

## 1. Introduction

Next-generation sequencing (NGS) has revolutionized genome sequencing owing to its massively parallel sequencing capacity and analytical sensitivity. This has made simultaneous screening of multiple markers on multiple samples feasible to support routine genome sequencing applications meant for either research or clinical use. Consequently, these technologies have reached unprecedented levels of acceptance and implementation and are actively replacing classical technologies, such as Sanger sequencing, hybridization-based arrays, fluorescence in situ hybridization, qualitative real-time PCR, and PCR-coupled with fragment analysis platforms [1]. NGS can also simultaneously screen for a variety of genomic alterations, such as mutations, insertions, deletions, gene copy number alterations, and chromosomal translocations, as well as the methylation status [2]. NGS can be used for different scales of genome sequencing, including screening of specific genomic regions or markers (targeted genome sequencing; TS), areas encompassing the coding regions of the genes or all exons (whole-exome sequencing), and the entirety of the genome (whole-genome sequencing). The majority of clinical applications that screen for mutations in markers of established clinical significance utilize the targeted sequencing approach of NGS. This requires targeted enrichment of genomic regions of interest (ROIs) from the expansive background of the entire genome. This is a critical step to ensure that the application of NGS is aimed to sequence the markers of interest accurately and efficiently. It is an integral part of any targeted NGS workflow being applied in a wide variety of fields, such as population studies, sequencing of ecological specimens, study of evolution, and characterization of infectious diseases [3,4,5,6]. With an emphasis on applications for oncology, this review will summarize and evaluate various approaches and technologies being used for the targeted enrichment of sequences for the routine sequencing of tumors.

Targeted enrichment for sequences includes their focused augmentation thousands of fold from the whole genomic background, ensuring that they are predominantly represented in the sequenced DNA, with high specificity so they can be used to identify sequence variations [7]. This is necessary to ensure that the sequencing capacity is utilized for targeted sequencing of ROIs at a sufficient sequencing level or depth to confidently identify variants of interest while decreasing the risk of incidental findings with potential ethical complications [8]. This sequence enrichment is generally achieved by using two major approaches, namely PCR-based amplicon and hybrid capture-based methodologies [9]. A detailed account of these two approaches, their different variations, and their advantages and disadvantages will be discussed.

## 2. Amplicon-Based Target Enrichment

**Figure 1:**
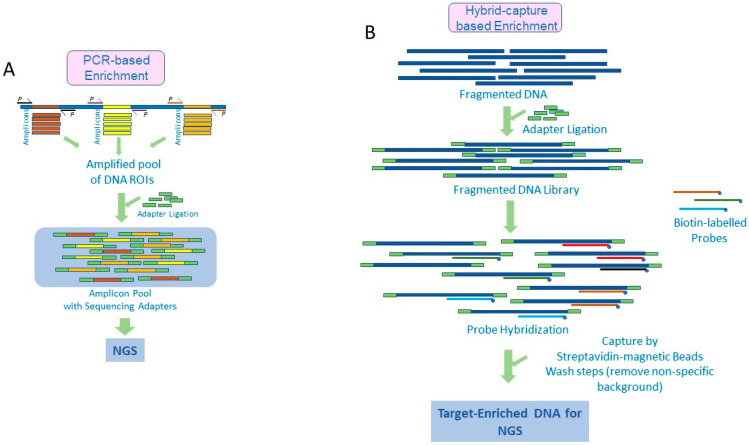
Target Enrichment Approaches for NGS. The underlying principles of the two prominent target enrichment technologies are depicted schematically. (**A**) The PCR amplicon-based method employs PCR amplification using sequence-specific, target-spanning primers to amplify and enrich the genomic regions of interest (targets). Postamplification, in a ligation step, sequencing adapters and barcode indexes are included to generate the genomic library for sequencing. (**B**) In the hybrid capture method, the input genomic DNA is fragmented using enzymatic or acoustic methods followed by ligation of adapters and barcode indices to generate a genomic library. This is subjected to hybridization using biotin-labeled target sequence-specific probes followed by capture of the hybridized probes using Streptavidin magnetic beads to isolate genomic regions of interest for sequencing.

The amplicon-based method involves amplification of the genomic ROI using polymerase chain reactions (PCRs). Here, primers are designed targeting areas flanking desired genomic regions and are used to amplify these regions several thousand-fold, thus enriching them for sequencing. Generally, multiple primers are designed, and PCR conditions are standardized so they work simultaneously in a single multiplexed PCR reaction to amplify all the target genomic regions. To the PCR products or amplicons, sequencing adapters (sequencing platform-specific synthetic stretches of DNA) are attached by ligation to generate a ‘library’ of the enriched DNA population ready for sequencing. These adapters include sequences utilized for clonal amplification of DNA prior to sequencing, barcode sequences that act as identification tags for the samples, and sequencing primer binding regions [10]. A schematic representation of the processes is presented in Figure 1A.

As PCR amplification is the basis of target enrichment in this method, it has proven to be very successful in sequencing applications where the nucleic acids are limited, in both quantity and quality. Consequently, this approach has thus become especially popular for tumor specimens that have limited sample size and cellularity or have been processed under harsh conditions (e.g., formalin fixation with paraffin embedding and FFPE or tissue decalcification using acid). As NGS screening generally involves multiplexed screening of numerous genes, PCR enrichment will require hundreds to thousands of primers to work in unison under similar PCR conditions [11,12]. To facilitate this, the careful design of the primers, their relative concentrations in the pool, and PCR conditions need to be established to ensure uniform amplification across all genomic ROIs. To this end, various design tools have been published [13,14]. Several commercial vendors (e.g., ThermoFisher Scientific (Waltham, MA, USA), Qiagen (Hilden, Germany), Fluidigm (San Francisco, CA, USA), and Integrated DNA Technologies (Coralville, IA, USA) also offer PCR-based enrichment methodology in their NGS library preparation kits, either as predesigned primer pools targeting specific panel of genes, or as custom design options. The predesigned panels have the advantage of being tested and validated by the vendor to provide a ready-to-use option. On the other hand, custom designs may need testing and fine-tuning to ensure successful and uniform enrichment of all ROIs.

PCR technology has evolved significantly over the years, and variations in designs have provided additional advantages. This evolution has tremendously expanded the efficiency and applications of PCR for nucleic acid characterization. These improvements also have been tested and implemented for NGS target enrichment, providing a wide variety of workflow options, for instance, long-range PCR that includes specialized PCR conditions and polymerases in order to amplify long regions of DNA (3–20 kb in length) [15,16]. This increased amplicon length provides several advantages, including requiring a smaller number of multiplexed primers to cover the ROI and providing greater uniformity of amplification and sequencing. This approach has been applied to sequence targets implicated in hereditary cancers, such as *BRCA1/2* [17]. Furthermore, technological improvements in PCR, such as droplet PCR—where the PCR reaction is compartmentalized into individual droplets, each acting as a microreactor for PCR—has also been used for NGS target enrichment. Here, the enrichment reaction is partitioned into millions of droplets, thus performing numerous parallel PCR enrichment reactions. Although this method requires investment in additional instrumentation to generate the droplets, amplification, and isolation of the target-enriched library for sequencing, it facilitates the use of large numbers of primers while minimizing undesirable interactions among them, ensuring uniformity of target enrichment across all ROIs [10,18,19,20].

Another method of compartmentalization of the enrichment reaction uses microfluidics, where parallel fluidic delivery of the DNA and PCR mix to an array of miniaturized reaction compartments used for simultaneous amplification of the targets in multiple samples. This technology provides the advantages of minimizing the DNA and reagent requirement to nanoliters along with automation and consolidation of the PCR reaction onto one nanofluidic chip. Post-PCR, the amplicons from each sample can be retrieved from the reaction compartments and processed further for sequencing [21,22]. Another specialized PCR technology that has been adapted for NGS target enrichment is anchored multiplex PCR, where the amplification is open-ended: only one side of the ROI sequence is targeted using a target-specific primer (anchor), while the other end is targeted with a universal primer. To facilitate this, universal adapters are ligated to double-stranded cDNA or DNA, and the ROI is amplified using the anchor primer at one end and the universal adapter-specific primer at the other end. This contrasts with the conventional PCR, where knowledge of both the 5′ and 3′ sequence is required to design primers for amplification. The ability of anchored PCR to work with one target-specific primer is significantly advantageous in decreasing the total number of primers necessary to amplify ROIs thus minimizing primer interference in the pool. It is also specifically useful in detecting fusions of any gene of interest without prior knowledge of its fusion partner, which has been very useful in the screening and detection of novel fusions [23,24,25].

COLD-PCR (co-amplification at lower denaturation temperature PCR) is another PCR modification that has been implemented for target enrichment prior to NGS. This technique specifically enriches the variant-harboring DNA strands by inducing formation of heteroduplexes between the wild-type and variant DNA strands and using their lower melting temperatures to specifically denature and amplify them further. In this way, the wild-type DNA strand background is progressively diminished, and variant-containing strands are selectively enriched [26,27]. When applied to NGS, this technology is very helpful in enriching the DNA library of all variants, including low-level mutations (2–5% variant allelic frequency). This facilitates a higher detection capacity and confidence for low-level mutations without the need for very deep sequencing, thus enhancing the accuracy and cost-efficiency of NGS [28]. In addition to these PCR variations, a modified version of amplicon-based target enrichment known as the extension–ligation-based method has also been implemented for NGS. Here, complimentary probes tagged with universal primer binding regions are used to bind either side of the genomic ROI. One of the probes is then extended to the second probe, thus generating a strand complementary to the ROI. Employing these as templates, universal primers are used to amplify the ROI and to add barcodes, sequencing adapters, and sequencing primer binding regions to generate the sequencing library [29].

Overall, owing to their versatility, compatibility with challenging specimens, and relatively fast and simple workflows, the PCR amplicon-based methods are preferred and applied for NGS target enrichment.

## 3. Target Enrichment by Hybrid Capture

In this method, genomic ROIs are enriched by employing sequence-specific, single-stranded oligonucleotides, referred to as ‘baits’ or ‘probes.’ These probes, which could be RNA or DNA, are hybridized to the RIOs and subsequently isolated to capture the target genomic sequences. In comparison to DNA baits, RNA baits provide better hybridization specificity and higher stability when bound to the DNA ROIs, thus increasing the overall efficiency of hybrid capture [30]. However, due to the relatively labile nature of RNA and the additional care necessary in the storage and use of RNA probes, DNA probes are predominantly in use.

Generally, the hybrid capture workflow involves fragmentation of DNA by either sonication (acoustic shearing) or enzymatic cleavage, followed by denaturation and hybridization with biotin-labeled capture probes. In order to amplify the starting genomic input before hybrid capture, synthetic DNA adapters are ligated to the fragmented double-stranded DNA, and adapter-specific primers are used to PCR amplify the total genomic DNA. The resulting genomic library is denatured and subjected to hybridization with the oligonucleotide probes, which bind to their complementary sequences in the ROIs and help in their selective capture. Depending on the mode in which the probes are presented to the target DNA, there are generally two methods of hybrid capture. The first is the ‘solid phase’ capture method, where the probes are immobilized on a solid surface, such as a glass slide, and the sample DNA is hybridized to it [31]. The second is the ’solution capture’ method, where the probes and denatured sample DNA library are allowed to interact in a solution for target capture [32]. Solid phase capture methods were the earliest target enrichment methods to be developed and adapted; however, owing to their workflow complexities, such as the need for specialized and expensive equipment for hybridization and limitation in terms of the number of samples that can be processed at a time (throughput), they are no longer preferred. Consequently, solution capture methods are the preferred methods currently and will be the focus of this review.

In the solution capture method, after the hybridization, the probe-bound genomic ROIs are captured using magnetic streptavidin beads, and any nonspecific genomic background bound to the beads is eliminated by several stringent washing steps. The captured ROIs are then isolated by eluting from the streptavidin beads, following which a second PCR step, using the adapter-specific primers, is run, which further amplifies the enriched DNA before sequencing [7,10]. The major steps in the workflow are represented in Figure 1B.

Several factors in the solution capture workflow can affect the efficiency of hybrid capture. One of the important factors is the extent of fragmentation of DNA before hybridization. This must be controlled to make sure that the average fragment size is appropriate to the intended sequencing length. A shorter fragment length will ensure higher specificity of capture, whereas larger fragment lengths will generally lead to a higher degree of off-target sequence captured. However, longer fragment lengths can be advantageous in increasing the confidence of alignment to the reference sequence and minimizing overrepresentation of the probe side of the sequence. Another important factor is the PCR amplification of the library, prior to hybridization, using adapter-specific primers. If used in excess, this step can selectively enrich the library with certain sequences, leading to decreased uniformity of coverage. This PCR step might be unavoidable when dealing with samples that yield low quality and quantity of DNA, such as tumor samples. When used, it is recommended to minimize the prehybridization PCR cycles, use blocker adapters during hybridization to minimize undesirable interactions of the adapter sequences with the sample DNA, and use PCR post-hybridization to amplify the captured target library prior to sequencing [10,33]. The time of hybridization also has a significant effect on the efficiency of target capture and is dependent on the methodology, complexity, and size of the target ROI, sample type, and the DNA quantity and quality. The hybridization time can vary between 2 and 48 h and needs to be established by preliminary feasibility studies for the design of each NGS panel and methodology used to identify the shortest hybridization time that can provide the maximum target enrichment [34,35,36].

**Figure 2:**
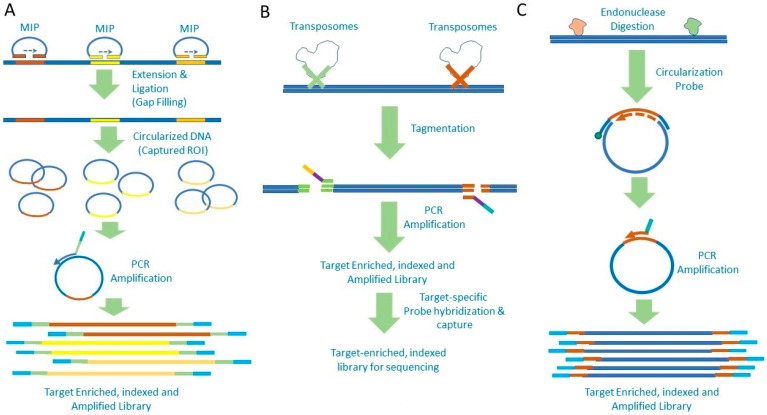
Variations of the Hybrid Capture Target Enrichment Methods. Some examples of variations of the hybrid capture methods are depicted. (**A**) Molecular inversion probe-based enrichment, where probes with sequences complementary to the target region of interest are used. Binding of the probes, followed by extension ligation results in circularized DNA with the target region included. Using these as templates, subsequently, a universal primer is used to linearize, amplify, and generate the sequencing library. (**B**) Tagmentation method, where transposomes are used to fragment double-stranded DNA and tag with sequencing adapters in a single step. This tagged genomic library is amplified using adapter-specific primers followed by hybrid capture with target-specific probes to isolate target-enriched library for sequencing. (**C**) Haloplex method, where biotin-labeled circularization probes are used to bind to endonuclease-digested DNA, followed by extension ligation, which results in circularized DNA with targeted region of interest, which is amplified using a universal primer to generate enriched library for sequencing.

In addition to the typical methodology of the hybrid capture workflow, several variations have been designed and implemented for NGS (schematically depicted in Figure 2). In one such variation, the target capture includes the use of molecular inversion probes (MIPs). These are single-stranded oligonucleotides with stretches complementary to the target sequence at both ends of the probes joined via a linker sequence carrying the site for universal primer binding. Binding of the probe to the target sequence and subsequent extension and ligation result in circularization of the DNA while capturing the target sequence. Subsequently, rolling circle amplification using universal primers is performed to include the sequencing adapters and to linearize and amplify the genomic library for NGS (Figure 2A) [37]. This method provides a high level of specificity and has been applied to detect low-level mutations with high accuracy. Another method variation is the transposon-mediated fragmentation or tagmentation, in which transposomes are used to fragment the DNA and add adapter sequences in a single step (Figure 2B). Consequently, the overall process is very similar to the typical hybrid capture; however, multiple steps involved in the workflow (DNA fragmentation, A-tailing, and ligation) are replaced by a single step, which significantly increases the efficiency of the workflow. It also increases the yield of the sequencing library by abrogating the loss of the processed DNA library that occurs due to multiple steps in the typical hybrid capture workflow and decreasing the overall hands-on time for target enrichment [38]. Tagmentation has been further improved by using transposomes immobilized on microbeads, referred to as tagmentation on microbeads or TOMs, which facilitates the isolation of tagmented DNA. Furthermore, by using transposomes with barcodes, adjacent sequences can be labeled similarly, which helps in obtaining a 2- to 3-fold longer assembly of continuous sequence in comparison to the typical target enrichment processes. This also helps in minimizing amplification bias, and it facilitates the identification of gene copy number variation with higher efficiency [39].

Another method of interest is hybrid capture using CircLigase, an enzyme which can ligate the 5′ phosphate and the 3′ hydroxyl group of single-stranded DNA or RNA. This capability was exploited for the development of a unique library preparation method referred to as the ‘hook ligation’ or ‘hook–capture method’ [40]. Here, probes or baits referred to as ‘hook probes’ are annealed to denatured DNA, and the free 5′ phosphate of the probe is ligated to the 3′ hydroxyl group of the target DNA to create a double-stranded open structure of the probe and the target DNA. Subsequently, the second strand of the target DNA is synthesized by 5′ to 3′ of the annealed probe extension using a polymerase, followed by ligation of universal adapter sequences. An exonuclease step eliminates all single-stranded nucleic acids, and amplification of the library using universal primers results in a ready-to-use DNA library. Similar to tagmentation, this method not only abrogates several laborious steps in library preparation but also avoids the need to use biotin-labeled probes and streptavidin beads, which makes the overall process efficient and economical for routine use.

Another variation of the hybrid capture is the Haloplex method (Agilent Technologies, Santa Clara, CA, USA), where hybrid capture is mediated by biotin-labeled circularization probes. The probes have the universal primer binding site, sample indices, and adapters necessary for sequencing sandwiched in between two stretches of sequences complementary to the ROI. DNA is fragmented by endonuclease digestion and subjected to hybridization to the probes, followed by extension and ligation of the DNA strand. This results in circularized DNA of the captured ROI along with the universal primer binding site. Probe-bound DNA is isolated using streptavidin beads, after which the universal primer is used to amplify and linearize the library DNA for sequencing (Figure 2C) [41,42].

## 4. Automation of Target Enrichment

Most target enrichment approaches have multiple steps involving varied processes, such as fragmentation, adapter ligation, PCR amplification, hybrid capture, and several DNA clean up steps. Performing them manually and routinely tends to carry the risk of workflow inconsistencies, cross contamination, and significant investment of hands-on time. Considering the complexity, high time consumption, and cost of the NGS library preparation reagents, automation of these steps is highly recommended, especially in situations where large numbers of samples are processed routinely. Several liquid handling and pipetting platforms have been tested and applied to automate these steps. Several solutions from well-known vendors have been tested and implemented, such as NGS Star and Starlet (Hamilton, Reno, NV, USA), Bravo (Agilent), Freedom EVO NGS (Tecan, Männedorf, Switzerland), epMotion (Eppendorf, Hamburg, Germany), KingFisher Duo and AB Library Builder systems (ThermoFisher), Biomek platform (Beckman Coulter, Brea, CA, USA), and JANUS, Sciclone and Zephyr platforms (Perkin Elmer, Waltham, MA, USA) [43]. Most of these platforms provide predesigned protocols for commonly used library preparation methodologies, along with varied levels of flexibility for designing and modifying the deck and the protocols to suit the laboratory’s needs. They also provide capabilities to integrate additional devices to vary the level of automation as required. Some of the liquid handlers, such as Mosquito HTS, are designed to process very low reagent volumes with high accuracy, which helps minimize reagent utilization [44].

In addition to liquid handlers, microfluidic approaches for library preparation allows for significant levels of automation. They involve varied approaches, such as droplet microfluidics, where the PCR amplification, barcoding, and library preparation occur in microscopic droplets, which act as microreactors [18,43], and multilayered soft lithographics microfluidics systems, which use microfluidics in a cartridge to miniaturize and conduct parallel library preparations on several samples simultaneously [45]. Despite the initial investment in instrumentation for automating the NGS library preparation workflow, the associated advantages of increased efficiency, minimized possibility of contamination, and decreased hands-on time justify the incorporation of these automated workflows into laboratories.

## 5. Comparison of Target Enrichment Methodologies

The two major types of target enrichment approaches of PCR or amplicon-based and the hybrid capture technologies have a fundamentally different methodology to capture and enrich the ROIs for NGS. This results in intrinsic differences in their advantages and disadvantages, and care needs to be taken by the user to preferentially choose between these two, considering several prerequisites, such as sample or tissue type, sample fixation and preservation method, expected quality and quantity of nucleic acid yield, and the type and level of genomic alteration being investigated. An overall summary of the major differences between these two methodologies along with examples of some of the commercially available enrichment options are presented in Table 1.

Numerous variations of these two fundamental methodologies have also been designed and tested by many laboratories in order to fine-tune them to suit their specific requirements. Consequently, a wealth of information is published and available for the readers to understand their strengths and weaknesses and make an informed decision to select the most optimal method. For instance, in one of the published studies, three different enrichment approaches were compared (microfluidics PCR; Fluidigm Access array, hybridization capture; SureSeq Solid Tumour hybridization panel by Oxford Gene Technologies (Begbroke, UK); and Ion AmpliSeq Cancer Hotspot panel; ThermoFisher Scientific) for their performance in detecting 24 mutations in six genes using DNA from FFPE and fresh samples of eight human bladder cancer cell lines [46]. DNA from FFPE samples was sequenced using all three enrichment strategies, whereas fresh DNA from the samples was sequenced using Fluidigm and SureSeq panels for comparison. Results for FFPE DNA showed that the SureSeq panel detected each of the expected 24 mutations (100% concordance), whereas the Fluidigm and the AmpliSeq panels were able to detect 20 and 18 mutations, respectively, indicating 83% and 75% concordance. In contrast, for fresh DNA, both Fluidigm and Sureseq panels detected each of the 24 mutations, indicating complete concordance. The main discordances arose due to the difference in the efficiency of the enrichment methods in dealing with FFPE samples, mutations being outside the ROI enriched in the panels, and sequencing drop-offs in a subset of genomic ROI. In another study, four different hybrid capture enrichment methods were compared to detect single nucleotide variants and copy number variations in a panel of 257 cancer-related genes [47]. The methodologies were SureSelect and Haloplex target enrichment (Agilent Technologies), Nextera (Illumina Inc., San Diego, CA, USA), and SeqCap EZ (Roche Nimblegen, Pleasanton, CA, USA), and sequencing was performed on MiSeq sequencer (Illumina Inc.). Of these methods, SureSelect uses RNA hybridization probes, whereas the rest use DNA probes. Sequencing uniformity, on-target sequencing rate, library complexity, and ability to detect single nucleotide variants and copy number alterations were compared. Overall cost was comparable across the methodologies, whereas the overall DNA requirement and workflow time were lower with Haloplex and Nextera when compared to the other two methodologies. Furthermore, methods involving DNA sonication (SureSelect and SeqCap) showed better library complexity and overall uniformity of sequencing coverage (including in areas of high and low GC%) in comparison to the methods that use enzymatic digestion of input DNA (Haloplex and Nextera). There was reasonable agreement in the single nucleotide variant (SNV) calls across all methods, and most of the discordances could be attributed to lack of sequencing coverage or failed regions of sequencing. Furthermore, Haloplex showed a pattern of unique SNV calls, predominantly in motifs prone to sequencing errors in Illumina sequencing chemistry, and it also missed some SNVs near the restriction enzyme digestion sites. A high degree of correlation was also observed across the methodologies for copy number variations. A similar comparison of the performance of four target enrichment methods (SureSelect, Haloplex, SeqCap EZ, and Ampilseq) as applied to the whole-exome target capture has also been reported [48]. Here, whole-exome capture libraries prepared using SureSelect, Haloplex, and SeqCap were sequenced using HiSeq2000 sequencer (Illumina Inc.), and the library prepared using AmpliSeq was sequenced by Ion Proton System (ThermoFisher Scientific). Comparisons were made with respect to sequencing uniformity, on-target sequencing rate, and capability to detect sequence variants (SNVs, indels, and CNVs). The evaluation found that hybrid capture-based methods (SureSelect and SeqCap EZ) performed better than amplicon-based methods (AmpliSeq and Haloplex) with regards to sequencing complexity, uniformity of coverage, and analytical sensitivity and specificity, indicating the ability to better call true positives and eliminate false positives. A similar conclusion was reached by another study, which tested the sequencing of *BRCA1/2* from FFPE tumor samples using a hybrid capture-based library preparation (TruSight, Illumina Inc.) and multiplex-PCR amplicon-based method (TruSeq custom amplicon; Illumina Inc.) [49]. Sequencing was performed using an MiSeq sequencer (Illumina Inc.), and the ability to detect SNVs, Indels, and CNVs in *BRCA1/2* genes was evaluated. The amplicon-based enrichment resulted in uneven sequencing coverage with failed sequencing in several regions, compromising accurate detection of mutations and CNVs. Additionally, the amplicon-based method also showed a higher false-positive rate due to the occurrence of sequencing artifacts. In contrast, the hybrid capture-based methodology provided more uniform coverage, less sequencing drop-off, and better analytical sensitivity due to more accurate detection of mutations and CNVs. Similar comparisons of these two methodologies for sequencing circulating cell-free tumor DNA or ctDNA have also been performed. Screening of ctDNA represents a revolution in molecular testing of tumors, where ctDNA isolated from blood plasm is used to screen for oncology markers [50]. Target enrichment from ctDNA is more challenging in comparison to other tumor sample types owing to the compromised quality and quantity of tumor-specific DNA isolated from plasma and the need for higher sensitivity and specificity of mutations detection. In a representative study, a comparison of ctDNA sequencing to detect mutations was performed using the Avenio ctDNA expanded panel (hybridization-based capture, Roche) and QIAseq Human Comprehensive Cancer panel (Amplicon-based, Qiagen), with deep sequencing performed using NextSeq 550 sequencer (Illumina Inc.) [51]. Several parameters were evaluated, including sequencing coverage, coverage of clinically relevant variants, specificity and sensitivity of target enrichment, and their mutual concordance. Both the methods were efficient in detecting clinically relevant mutations with high sensitivity. The Avenio ctDNA workflow involved more preparation time (3 days), whereas the amplicon-based QIAseq workflow could be completed in one day. This difference was predominantly due to the prolonged hybridization step in the Avenio method (18 h), whereas the actual hands-on time for both methods was comparable. The Avenio ctDNA panel performed better in terms of successful capture of targeted ROI (99% vs. 85% for QIASeq) and analytical sensitivity, as determined by the concordance of the expected vs. detected variants (92.3% vs. 86.4% for QIAseq) for variants at allele frequencies of ≤20%. This difference was more prominent in detection of low-level variants (allele frequency of ≤5%) with the concordance of 75.0% (Avenio) vs. 53.8% (QIAseq), which indicated better mutation detection sensitivity of Avenio for low-level ctDNA mutations. In addition to the example publications discussed above, numerous publications have examined the PCR amplicon and hybrid capture-based methodologies for various applications and provide a valuable resource for laboratories in search of a target enrichment protocol. A summary comparison of the commercially available enrichment options is provided in Table 2. Overall, both PCR- and hybrid capture-based technologies are applicable for NGS target enrichment. However, hybrid capture-based methods are preferable owing to their better overall performance, despite some advantages the amplicon-based method offers, such as compatibility with low quality and quantity of nucleic acid input and quicker and less complex workflows.

To summarize, target enrichment for genomic regions of interest represents a critical step in targeted NGS sequencing workflow and can determine the success, efficiency, and accuracy of variant detection. Based on the mode of target enrichment, there are two major target enrichment methodologies (PCR amplicon-based and hybrid capture-based). Their extensive use, evaluation, and modifications over the years have resulted in numerous different approaches and protocols fine-tuned for specific applications. This represents a wealth of information and options that users should carefully consider when deciding on a target enrichment technology to suit the needs of their laboratory.

## Figures and Tables

**Table 1:** Comparison of Amplicon-Based and Hybrid Capture-Based Target Enrichment. A comparison of the principles, workflow, overall performance, and commercially available options is provided.

	Hybrid Capture-Based Enrichment	PCR Amplicon-Based Enrichment
**Enrichment Principle**	Hybridization capture-based enrichment using single-stranded DNA or RNA probes complementary to the genomic regions of interest	PCR-based amplification using sequence-specific primers flanking genomic regions of interest
**Nucleic Acid Input**	Requires relatively high quantity of nucleic acid input	Compatible with low quantity of nucleic acid input
**Nucleic Acid Quality**	Compatible with challenging sample types; however, success depends on obtaining sufficient yield	Compatible with challenging sample types (e.g., FFPE and decalcified samples)
**Fragmentation of Nucleic Acid Input**	Required. Nucleic acids need to be enzymatic-digested oracoustically sheared prior to hybrid capture	Not required
**Workflow Time**	Relatively long due to the hybrid capture step	Significantly shorter workflow
**Workflow Complexity**	High complexity workflow with multiple steps	Relatively simple workflow
**Gene Targets per Panel**	No limitations and can include any number of gene targets. Preferred methodology for large panels and whole-exome sequencing.	Generally suited for smaller number of gene targets. Limited by the multiplexing capability of the primers
**Uniformity of Sequence Enrichment**	Higher uniformity of target enrichment and lower rates of sequencing failures in regions of interest	Relatively low target enrichment uniformity and higher sequencing failures
**Off-Target Sequencing Rate**	Relatively high. Has more possibility of off-target sequences captured and sequenced	Lower off-target sequencing rate
**Commercial Options**	SureSelect (Agilent Technologies) Haloplex (Agilent Technologies) XGen NGS Hybrid capture (Integrated DNA Technologies) TruSight Hybris capture (Illumina Inc.) Swift Hybrid Capture (Swift Biosciences, Ann Arbor, MI, USA)	AmpliSeq (ThermoFisher Scientific) AccesArray (Fluidigm Corporation, South San Francisco, CA, USA ) GeneRead (Qiagen) RainStorm (RainDance Technologies, Lexington, MA, USA) TruSeq (Illumina Inc.) HEAT-Seq (Roche) XGen NGS Amplicon Sequencing (Integrated DNA Technologies) Accel-Amplicon (Swift Biosciences)

**Table 2:** Comparison of Commercial Amplicon-Based and Hybrid Capture-Based Target Enrichment Methods. A snapshot summary of some published studies comparing commercial target enrichment products, their vendors, enrichment approaches, workflow, samples tested, and their overall performance is provided.

Commercial Enrichment Methods Compared	Enrichment Approach	Genome Targets and Sample Types	Findings	Reference
Fluidigm Access array (Fluidigm)	Microfluidic PCR	DNA from 8 human bladder cancer cell lines (both fresh and formalin-fixed samples). 24 mutations in 6 genes (*BRAF, FGFR3, KRAS, NRAS,* *PIK3CA,* and *TP53)* Fresh and formalin-fixed and paraffin-embedded DNA samples.	Complete concordance of results for fresh DNA SureSeq panel performed the best followed by Fluidigm and IonAmpliseq	[46]
SureSeq panel				
(Oxfore Gene Technology)	Hybrid-capture			
Ion AmpliSeq				
(ThermoFisher Scientific)	PCR amplicon-based			
SureSelect (Agilent Technologies)	Hybrid capture	Single nucleotide variants (SNVs) and copy number variations (CNVs) in a panel of 257 cancer-related genes. Cancer cell lines and tumor samples (breast, melanoma, lung, and colon cancer)	Comparable cost of workflow across the methods High level of concordance observed for SNV and CNV detection across methods SureSelect and SeqCap showed better library complexity and overall sequencing uniformity	[47]
Haloplex (Agilent Technologies)	Hybrid capture			
Nextera (Illumina Inc)	Hybrid capture			
SeqCap EZ (Roche Nimblegen)	Hybrid capture			
SureSelect (Agilent Technologies)	Hybrid capture	Whole-Exome Sequencing	Hybrid capture methods provided better library complexity, uniformity of coverage, analytical sensitivity, and specificity	[48]
Haloplex (Agilent Technologies)	Hybrid capture			
SeqCap EZ (Roche Nimblegen)	Hybrid capture			
Ion AmpliSeq				
(ThermoFisher Scientific)	PCR amplicon-based			
TruSight (Illumina Inc)	Hybrid capture	*BRCA1* and *BRCA2* sequencing FFPE tumor samples	TruSight performed better with regards to uniformity of sequencing, analytical specificity, and detection of mutations and CNVs	[49]
TruSeq custom amplicon				
(Illumina Inc)	PCR amplicon-based			
Avenio CtDNA panel (Roche)	Hybrid capture	Screening circulating cell-free tumor DNA (ctDNA) for cancer-related markers	QiaSeq had shorter workflow (1 day) in comparison to Avenio (3 days) Avenio fared better in enriching targets of interest (99% vs. 85% for QIASeq). Analytical sensitivity for Avenio (92.3%) was better than QiaSeq (86.4%). Detection sensitivity for low-level variants (≤ 5%) was better for Avenio (75%) vs. QiaSeq (53.8%)	[51]
QIAseq Human Comprehensive Cancer				
panel (Qiagen)	PCR amplicon-based			
